# Towards the integration of animal‐borne instruments into global ocean observing systems

**DOI:** 10.1111/gcb.14902

**Published:** 2019-11-27

**Authors:** David March, Lars Boehme, Joaquín Tintoré, Pedro Joaquín Vélez‐Belchi, Brendan J. Godley

**Affiliations:** ^1^ Marine Turtle Research Group Centre for Ecology and Conservation University of Exeter Penryn UK; ^2^ ICTS SOCIB – Balearic Islands Coastal Observing and Forecasting System Parc Bit Palma de Mallorca Spain; ^3^ Sea Mammal Research Unit Scottish Oceans Institute University of St Andrews St Andrews UK; ^4^ IMEDEA (CSIC‐UIB) Mediterranean Institute of Advanced Studies Esporles Spain; ^5^ Instituto Español de Oceanografía Santa Cruz de Tenerife Spain

**Keywords:** animal‐borne instruments, Argo, global ocean observing system, marine vertebrates, multi‐platform ocean observation, operational oceanography, pinnipeds, satellite tracking, sea turtles

## Abstract

Marine animals are increasingly instrumented with environmental sensors that provide large volumes of oceanographic data. Here, we conduct an innovative and comprehensive global analysis to determine the potential contribution of animal‐borne instruments (ABI) into ocean observing systems (OOSs) and provide a foundation to establish future integrated ocean monitoring programmes. We analyse the current gaps of the long‐term Argo observing system (>1.5 million profiles) and assess its spatial overlap with the distribution of marine animals across eight major species groups (tuna and billfishes, sharks and rays, marine turtles, pinnipeds, cetaceans, sirenians, flying seabirds and penguins). We combine distribution ranges of 183 species and satellite tracking observations from >3,000 animals. Our analyses identify potential areas where ABI could complement OOS. Specifically, ABI have the potential to fill gaps in marginal seas, upwelling areas, the upper 10 m of the water column, shelf regions and polewards of 60° latitude. Our approach provides the global baseline required to plan the integration of ABI into global and regional OOS while integrating conservation and ocean monitoring priorities.

## Graphical abstract

Marine animals are increasingly instrumented with environmental sensors that provide large volumes of oceanographic data. By linking sampling gaps in ocean monitoring with marine animal distributions, this study provides new insights into using such sensors more efficiently to complement ocean observing systems at global and regional scales. With limited potential to monitor the deep ocean, animal‐borne instruments could fill the gaps in marginal seas, upwelling areas, the upper 10 m of the water column, shelf regions and polewards of 60° latitude. This contribution could benefit ocean research in reducing monitoring biases and improving our understanding of key oceanographic processes.

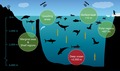

## 1 INTRODUCTION

Sustained and systematic observations of marine ecosystems are needed to understand how the ocean is changing both naturally and as a result of human activities (Hoegh‐Guldberg, 2010; Miloslavich et al., 2018). Actions towards a more integrated and sustainable ocean observing system (OOS) to facilitate ocean discovery and environmental monitoring are deemed essential for future progress (Cheng et al., 2017; Duarte, Poiner, & Gunn, 2018; Visbeck, 2018). International efforts, such as the Global Ocean Observing System (https://www.goosocean.org/), are contributing towards the integration of multiple platforms to monitor essential biodiversity variables (Miloslavich et al., 2018; Muller‐Karger et al., 2018), essential ocean variables (Lindstrom, Gunn, Fischer, McCurdy, & Glover, 2012) and estimate global ocean indicators, like global ocean heat content and global steric sea level (Von Schuckmann et al., 2014).

The ocean observation network experienced a revolution with the advent of the Argo array of profiling floats since the beginning of this century (Abraham & Baringer, 2013; Riser et al., 2016; Roemmich et al., 2019). Argo is comprised of a global array of free‐drifting profiling floats that measure temperature and salinity of the upper 2,000 m of the ocean (Riser et al., 2016). This project achieved its target of 3,000 active floats in 2007 providing a key component in the assessment of large‐scale ocean circulation and associated global ocean climate dynamics (Roemmich et al., 2019). However, despite the extensive coverage of the Argo network, large areas of the ocean still remain under‐sampled due to environmental (e.g. sea ice, shallow water, ocean divergence, wind drift), logistical (e.g. remote areas), political (e.g. exclusive economic zones [EEZ]) and security (e.g. piracy) reasons (Von Schuckmann et al., 2014). The main gaps include specific ocean regions such as the deep ocean at >2,000 m, high latitudes (>60°), the surface layer (<10 m), shelf regions and the marginal seas, including national EEZ (Von Schuckmann et al., 2016), which still leave systematic biases in the observation system and can have a large impact on the estimation of global ocean indicators (Henson, Beaulieu, & Lampitt, 2016; Von Schuckmann et al., 2014). Addressing these gaps is one of the key priorities identified in recent reviews of the OOS (Roemmich et al., 2019; She et al., 2019; Tanhua et al., 2019). The deep ocean and high latitudes are technological difficult regions to observe, but recent advances (e.g. Deep Argo programme) have enabled researchers to investigate environmental and even ecosystem processes (Fedak, 2013; Zilberman & Roemmich, 2017). The lack of data from the surface layer of the ocean is addressed using satellite data, voluntary observing ships and data from autonomous systems which can deliver high resolution data close to the ocean's surface (O'Carroll et al., 2019). This has helped to evaluate long‐term trends in eastern boundary upwelling systems (Sydeman et al., 2014); however, direct in situ observations are still necessary to evaluate the hypothesis suggested by Bakun (1990) that coastal upwelling intensification will occur in response to continued global warming. Marginal and shelf seas are particularly challenging due to navigational challenges to automated systems and potentially troublesome political issues (Riser et al., 2016). Recent technological advances with two‐way satellite communications (e.g. Iridium), and guidance on the use of, for example, floating buoys inside the EEZ of coastal states (IOC, 2008) has allowed the optimization of float endurances in marginal seas and coastal areas (Poulain et al., 2007; Roemmich et al., 2019). This has helped to clarify the details of water mass formation in the Mediterranean (Juza et al., 2019; Kokkini et al., 2019) and to improve predictions of the basin‐scale circulation by assimilating profile data into numerical models of the circulation (Nilsson, Dobricic, Pinardi, Taillandier, & Poulain, 2011). However, sampling inside EEZs is still challenging and requires major logistical and political support from coastal states (Hermes et al., 2019; Roemmich et al., 2019). Furthermore, there is a need to enhance coverage in critical areas such as tropical regions, with large influence on global climate variability and weather, and western boundary regions, with high levels of mesoscale variability (Roemmich et al., 2019; Smith et al., 2019). Therefore, the challenge is to advance towards sustained multi‐platform and integrated observing systems that allow systematic monitoring of the wide range of spatial and temporal scales of ocean circulation, from local to sub‐basin and global basin scale (Tintoré et al., 2013).

Instrumenting animals is not a new idea as it can be a useful tool to collect basic information (e.g. on animals' movements, physiology), to gain ecological and evolutionary insights, to assess species vulnerability to climate change and to project past, current and future species distributions (Boehme et al., 2012; Holloway & Miller, 2017; Kooyman, 1966; McMahon & Hays, 2006; Payne et al., 2018; Wiens, Stralberg, Jongsomjit, Howell, & Snyder, 2009). Furthermore, while this technology can provide key information on essential biodiversity variables (e.g. species distribution, physiology, movement, species interactions), animal‐borne instruments (ABI) can now also provide essential ocean variables such as temperature, conductivity, light level, oxygen and chlorophyll (Bailleul, Vacquie‐Garcia, & Guinet, 2015; Boehme et al., 2009; Coffey & Holland, 2015; Harcourt et al., 2019; Laidre, Heide‐Jørgensen, Logsdon, Delwiche, & Nielsen, 2010; Teo et al., 2009). Therefore, integrating ABI can complement ocean observing platforms such as Argo floats, gliders and other autonomous vehicles to provide unique and cost‐effective data from poorly sampled ocean regions (Block et al., 2016; Bograd, Block, Costa, & Godley, 2010; Fedak, 2004; Harcourt et al., 2019; Hays et al., 2016; Hussey et al., 2015; Roemmich et al., 2010; Roquet et al., 2014). For instance, ABI in the marine environment have been deployed on pinnipeds (Bailleul et al., 2015; Roquet et al., 2014), cetaceans (Laidre et al., 2010), marine turtles (Chambault et al., 2015, 2016; McMahon et al., 2005; McMahon & Hays, 2006; Patel et al., 2018), sharks (Coffey & Holland, 2015; Payne et al., 2018), fish (Block, Costa, Boehlert, & Kochevar, 2002), flying seabirds (Wilson et al., 2002; Wilson & Vandenabeele, 2012), penguins (Charrassin, Park, Maho, & Bost, 2002; Sala, Pisoni, & Quintana, 2017) and sirenians (Hagihara et al., 2018). Animals can travel to regions that are relatively inaccessible to other ocean observing technologies. For example, they can stay in areas in which passive platforms are often pushed away (i.e. upwelling zones; Block et al., 2016) or they have problems transmitting their data (e.g. sea ice zones; Nicholls, Boehme, Biuw, & Fedak, 2008). Previous studies using animal instruments have focused on polar areas, where pinnipeds are able to sample the upper 700 m in areas of ice cover that are inaccessible to conventional observing platforms (Fedak, 2013; Riser et al., 2016). In the polar regions, data provided by marine mammals have been used to study the biology of the species, analysing physical ocean processes and improving bathymetric data sets in regions lacking detailed sounding data (Fedak, 2013; Padman et al., 2010; Pauthenet et al., 2018; Pellichero, Sallée, Chapman, & Downes, 2018). Few studies, however, have been conducted regarding the use of animal oceanographers at mid and low (tropical) latitudes (McMahon et al., 2005; Patel et al., 2018), where significant gaps in global ocean monitoring still remain (Roemmich et al., 2019; Von Schuckmann et al., 2014).

Previous global syntheses have analysed the overlap of marine species distributions with human impacts (Selig et al., 2014; Tittensor et al., 2010), marine protected areas (Klein et al., 2015; O'Hara, Afflerbach, Scarborough, Kaschner, & Halpern, 2017) or geopolitical boundaries (Harrison et al., 2018) to identify priority conservation areas and guide international strategies for managing marine species. In this study, we analyse the gaps in coverage of long‐term OOSs and the overlaps with the distribution of multiple species to assess the potential role of ABI in contributing ocean research at global level. The recent development of standardized databases offers an unprecedented opportunity to link marine animal distribution across multiple taxa and operational OOSs at a global scale. The present study uses data from the long‐term Argo database and open global biogeographical databases (Halpin et al., 2009; IUCN, 2017; Treasure et al., 2017). First, we analyse the inter‐annual persistence of gaps of the Argo network to map under‐sampled regions and identify priority areas for ocean monitoring. Then, we overlap the under‐sampled regions with extent of occurrence (EOO) maps of marine vertebrates across multiple taxa to determine the potential locations where ABI could contribute to the Global Ocean Observing System (https://www.goosocean.org/). Finally, we focus on pinnipeds and marine turtles as they are often equipped with ABI and incorporate satellite tracking data into our analysis.

## 2 MATERIALS AND METHODS

### 2.1 Gap analysis of the Argo network

We assembled and analysed the Argo database (>1.5 million profiles, >13,000 instruments; Argo, 2000) to map un‐sampled and under‐sampled areas and assess the persistence of gaps over the 2005–2016 period. We identified Argo coldspots (i.e. spatial coherent structures larger than 25 square degrees with a gap persistence rate of ≥80%), and summarized them as function of latitude, bathymetry and political boundaries (see Methods S1).

### 2.2 Animal‐borne platforms

We compiled a comprehensive species list of 183 marine vertebrates which can be equipped with instruments across eight taxonomic groups using previous multi‐specific reviews (Hussey et al., 2015; Lascelles et al., 2016; Sequeira et al., 2018) and public databases (Halpin et al., 2009; Treasure et al., 2017). The list included those species that were equipped with any kind of instruments using satellite communications or cellular networks, in order to illustrate those species that have the current or future potential to relay near real‐time oceanographic data from remote locations. We represented their spatial distribution at a global scale using extent of occurrence (EOO) maps created from public available databases (BirdLife International, 2017; Halpin et al., 2009; IUCN, 2017; Kot et al., 2016). EOO maps were rasterized using extent areas and summarized by number of species per taxonomic group. A number of species values for each taxon were then normalized by rescaling from zero to one, and averaged across taxa by cell for all taxa (Tittensor et al., 2010). In order to compare with EOO maps, we analysed telemetry observations for pinnipeds (*n* = 10) and marine turtle (*n* = 7) species. These two taxonomic groups presented the highest number of satellite tracking studies (Hussey et al., 2015) and data for a large number of animals (>3,000) were available from public databases (Halpin et al., 2009; Treasure et al., 2017; see Methods S2). Maximum dive depths were extracted from public online databases (Froese & Pauly, 2019; IUCN, 2017; Palomares & Pauly, 2019; Ropert‐Coudert, Kato, Robbins, & Humphries, 2018) and previous reviews (Halsey, Butler, & Blackburn, 2006; Hochscheid, 2014; Ponganis, 2015). Maximum dive depths were not available for 61 species; hence, 122 of the 183 selected species were used to assess the potential vertical coverage.

### 2.3 Spatial overlap

We assessed the spatial overlap between species distributions (i.e. presence/absence from both EOO and tracking data) and Argo coldspots using two complementary indices:

(1) $${\text{OV}}_{\text{coldspot}}=S/C,$$

(2) $${\text{OV}}_{\text{range}}=S/R,$$

where *S* is the shared surface between the species range and the coldspot regions, *C* is the surface occupied only by the coldspots and *R* is the surface used only by the species. The first index, OV_coldspot_, represents the proportion of coldspots that are covered by the range of a single species. The second index, OV_range_, indicates the amount of one species range that overlaps with coldspots surfaces, and can be understood as an indicator of the specificity of such species to remain within coldspots areas. We calculated the spatial overlap indices for the global ocean (90°S–90°N) and five sectors of the world oceans limited by the 30th and 60th parallels (Von Schuckmann et al., 2014).

### 2.4 Data projection and representation

All spatial data sets were collated at 1° resolution and converted to the Mollweide projection with a WGS84 datum as it is an accurate single global projection that preserves geographic area and allows data transfer and analysis among operating systems and software (see Methods S3).

### 2.5 Data availability statement

The raw data that support the findings of this study are available from their corresponding data providers, but restrictions may apply to the availability of these data, which were used under license for the current study. Raw data are, however, available from the authors upon reasonable request and with permission of third‐party data providers. The new generated data sets from this study are available at Figshare (https://doi.org/10.6084/m9.figshare.7992572).

### 2.6 Code availability

All data processing and analysis were completed using R statistical software. All code is available on GitHub at https://github.com/dmarch/abigoos and DOI https://doi.org/10.5281/zenodo.2638123.

## 3 RESULTS

### 3.1 Gap analysis of Argo network

**Figure 1:**
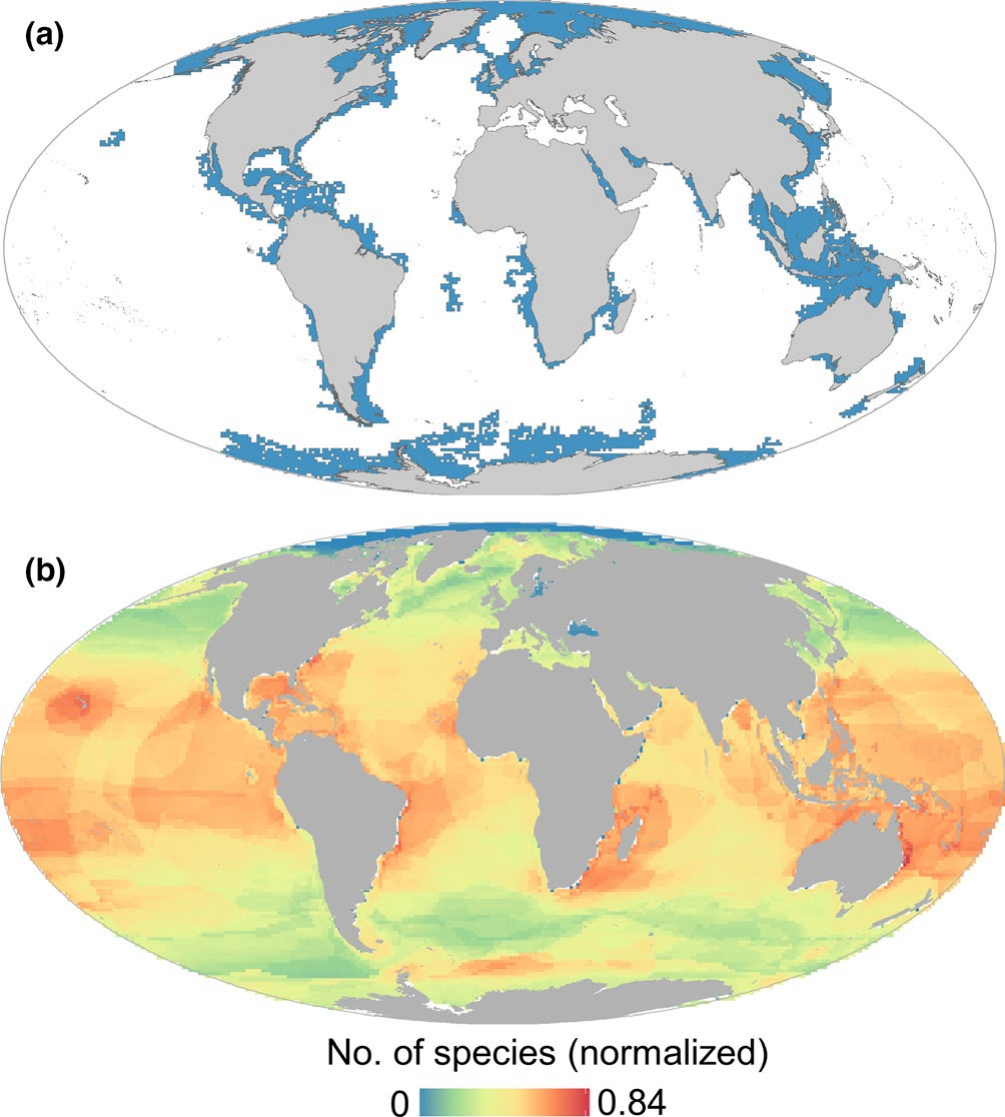
Global patterns of the spatial distribution of the Argo network and marine species subject to telemetry. (a) Argo coldspots. (b) Density map of marine species. Map created by overlaying the extent of occurrence maps for all identified species that were equipped with satellite/global system for mobile communications tags in previous studies. A number of species for each taxon were normalized by rescaling from zero to one and averaged across taxa by cell for all taxa

The spatial pattern of the Argo network reflects a global effort of ocean monitoring with a slightly higher concentration of profiles in the northern hemisphere (Figure S1a). Such a pattern is consistent throughout the years (Figure S2), presenting a higher inter‐annual variability at higher latitudes (>60°) and coastal regions (Figure S1b). Sampling gaps considered both un‐sampled regions (i.e. areas without profiles) and under‐sampled regions (i.e. areas pertaining to the lowest quintile, <20%, of the Argo profile density distribution). The sampling gap surface was mainly comprised by under‐sampled regions (>70%) and presented a slight decreasing trend throughout the years (Figures S3 and S4). Consequently, the spatial distribution of the sampling gap persistency (i.e. the percentage of years a cell was identified as sampling gap) is presented in Figure S1c. Only spatial coherent gap areas larger than 25 square degrees with a gap persistence rate of ≥80% were retained as coldspot areas (Figure 1a). The surface of the coldspot areas totals 69,760,000 km^2^, representing 18.6% of the estimated global ocean surface (Table S1). Coldspots were concentrated in higher latitudes (40.4% of the coldspot surface was found polewards of 60°), in tropical regions (37.4%) and shallow waters (34.2% at <200 m depth; Figure S5). In tropical regions, coldspots were mainly located in shallow waters, equatorial areas and marginal seas, such as the Caribbean or Indonesian Sea. Moreover, coldspots were mostly found within economic exclusive zones (EEZs, 75.7%). EEZs with higher coldspot surfaces corresponded to Russia, Indonesia, Antarctica, Canada and United States of America (Table S2).

### 3.2 Global distribution of potential animal‐borne platforms

**Figure 2:**
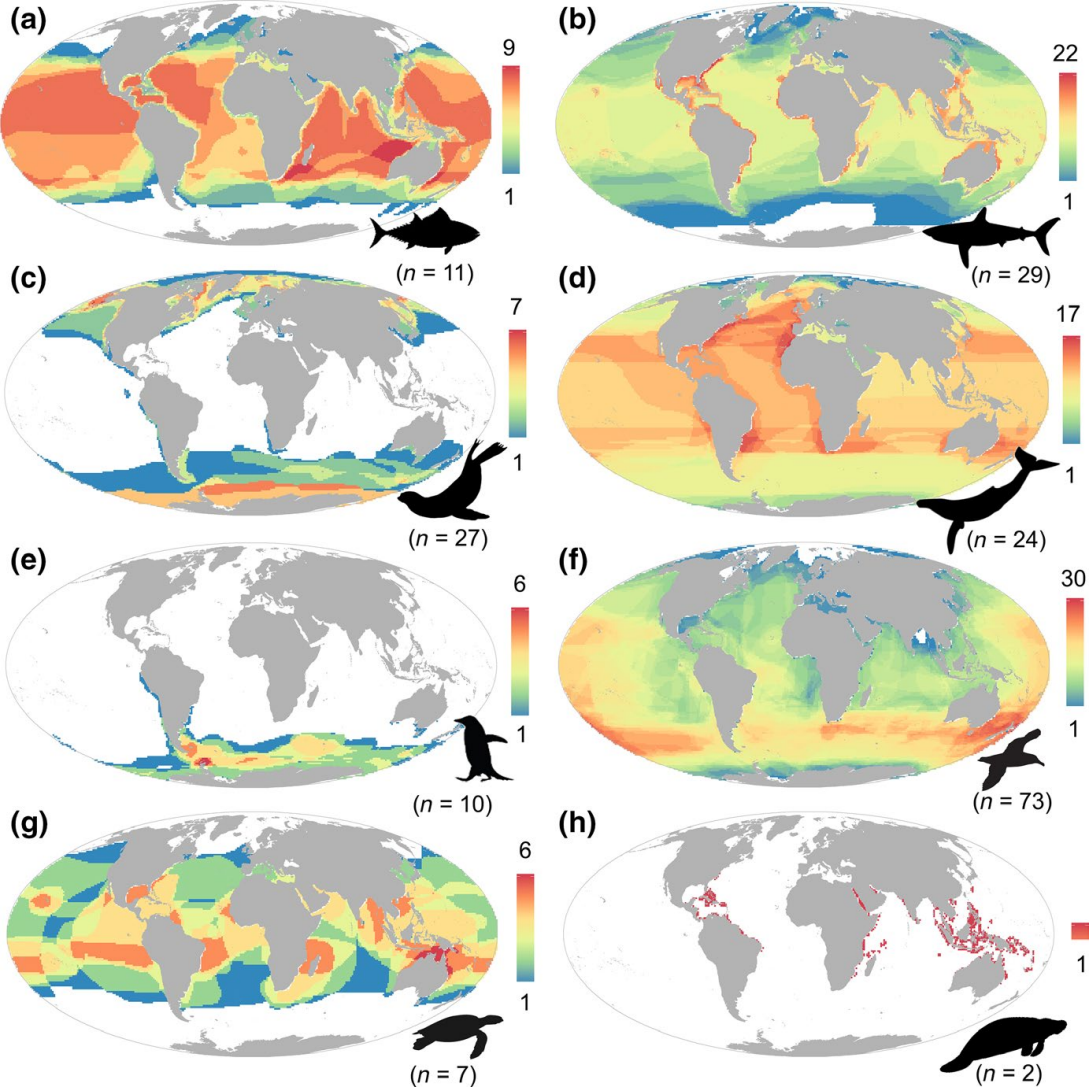
Density maps of species subject to telemetry by taxonomic group. (a) Tuna and billfishes, (b) sharks and rays, (c) pinnipeds, (d) cetaceans, (e) penguins, (f) flying seabirds, (g) turtles and (h) sirenians. Maps created by overlaying extent of occurrence maps for all identified species that were equipped with satellite tags in previous works. Colour scaling is adjusted by taxonomic group to optimize contrast. The total number of species per taxonomic group is indicated in parentheses

**Figure 3:**
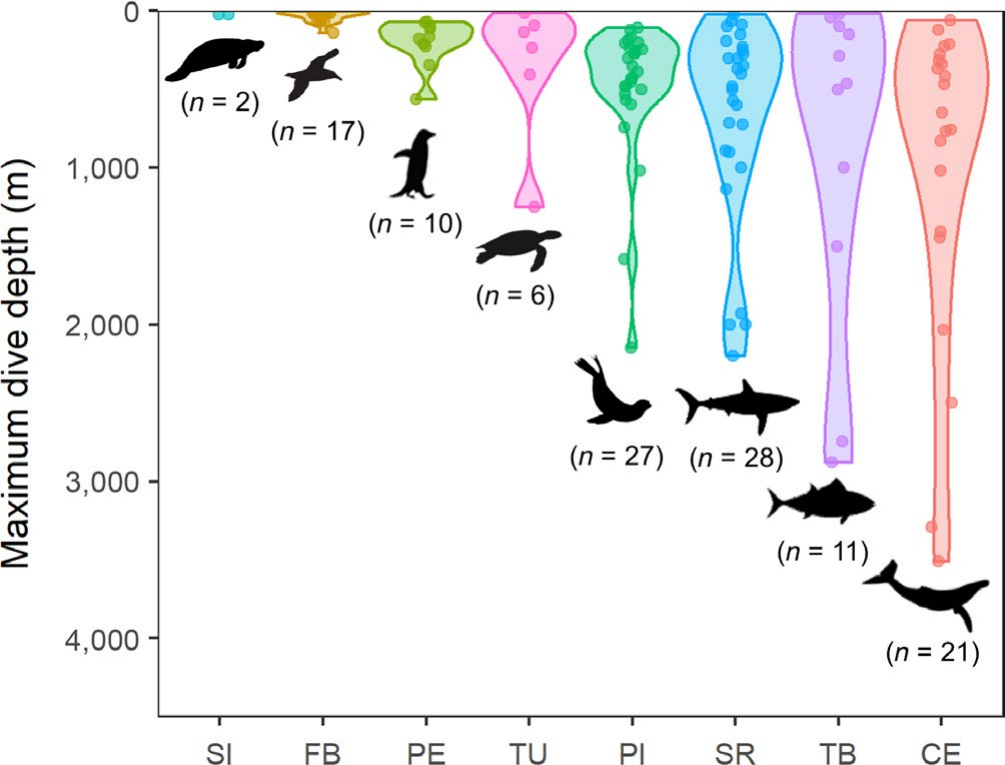
Maximum dive depths. Violin plot of maximum dive depths of species subject to telemetry by taxonomic group. CE, cetaceans; FB, flying seabirds; PE, penguins; PI, pinnipeds; SI, sirenians; SR, sharks and rays; TB, tuna and billfishes; TU, turtles. Total number of species per taxonomic group is indicated in parentheses

The selected species of potential animal‐borne platforms comprised a broad range of species across multiple taxa. The pooled distribution of all selected species resulted in higher densities at mid‐ and lower latitudes (Figure 1b; Table S1). Pinnipeds, flying seabirds and penguins peaked at higher latitudes, while the other groups dominate at mid‐ and lower latitudes (Figure 2). At the taxonomic group level, cetaceans and large bony fishes presented higher range sizes, whereas sirenians exhibited the smaller range sizes (Table S3). At the species level, EOO range sizes spanned from 140,000 km^2^ for the western gull (*Larus occidentalis*) to 367,920,000 km^2^ for the killer whale (*Orcinus orca*; Data S1). Information on maximum dive depths (Figure 3; Table S3) illustrates the potential of ABI to support vertical measurements. Most of the species analysed in this work (63.9%) could potentially support vertical measurements until 200 m depth (i.e. epipelagic zone), while only a small proportion (15.6%) showed the potential to profile the ocean down to 1,000 m (i.e. parking depth of Argo floats in global basins).

### 3.3 Overlap between animal‐based platforms and Argo coldspots

**Figure 4:**
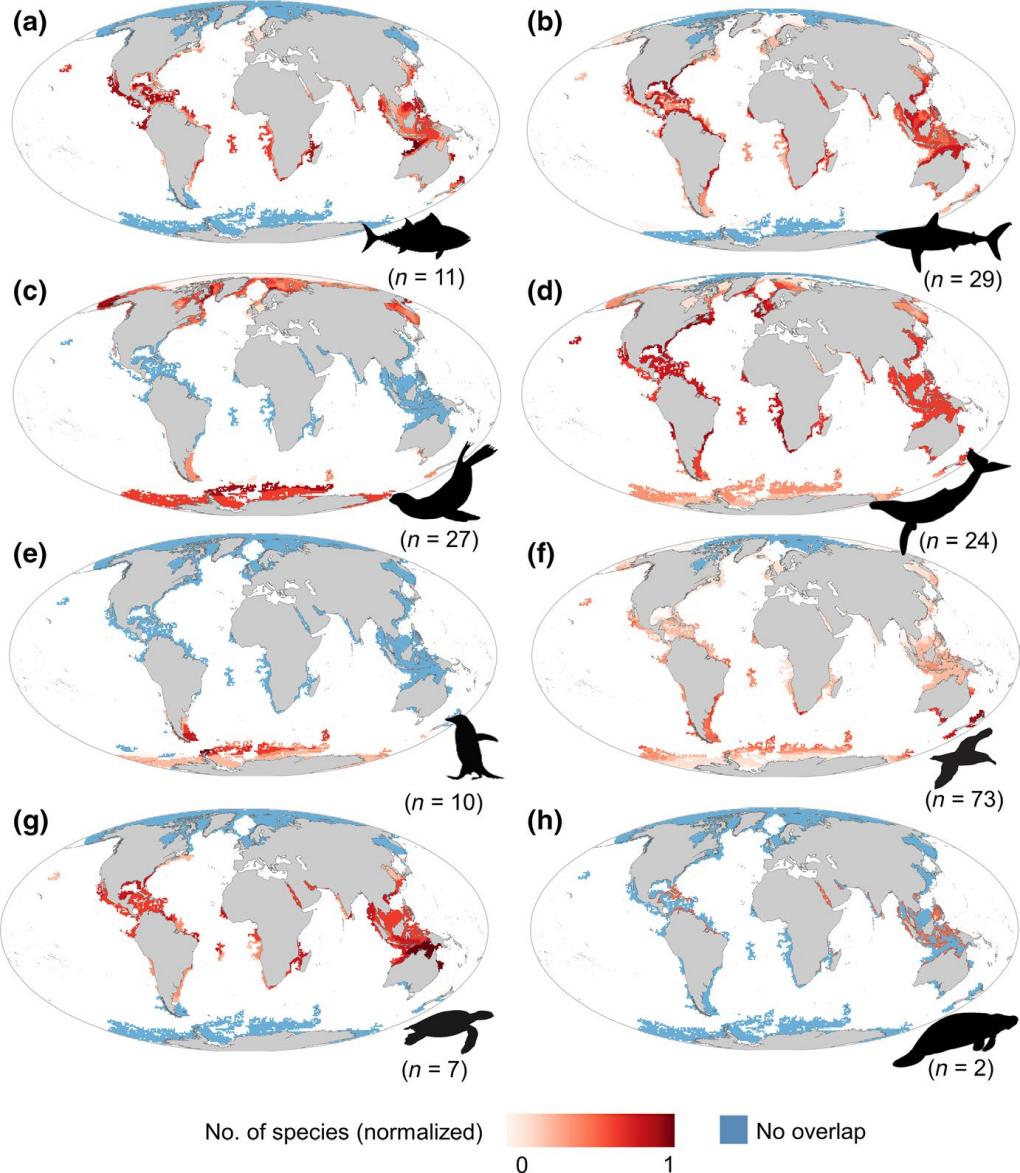
Overlap between gaps of the Argo network and marine species subject to telemetry. Maps represent the spatial overlap between gaps of the Argo network and extent of occurrence maps by taxonomic group: (a) tuna and billfishes, (b) sharks and rays, (c) pinnipeds, (d) cetaceans, (e) penguins, (f) flying seabirds, (g) turtles and (h) sirenians. Red cells represent the normalized number of species for each taxon and identify potential areas where animal‐based platforms could complement the Argos network. Blue cells indicate Argo gap areas where no species of animal‐borne platforms occur. Numbers between parentheses represent the number of species per taxonomic group

Overlap maps by taxonomic group illustrate the regions where animal‐based platforms could complement the Argo network at global scale (Figure 4). At higher latitudes, pinnipeds and penguins constitute the main groups that could complement coldspots, with some contribution of some species of cetaceans and flying seabirds (Figure S6). At mid‐ and low latitudes, the remaining groups present a higher overlap with coldspots. Together with sea turtles, cetaceans, sharks and rays and tuna and billfishes are present in most of the coldspot areas. The distribution range of sirenians limits the potential of this group to contribute to OOS in a small fraction of coldspots in tropical regions.

The overall overlap across all taxa with the Argo coldspots (i.e. OV_coldspot_) was shown to be higher than average in the Antarctic Ocean, followed by the Southern Ocean and Tropical Ocean (Table S1). The spatial overlap at species levels shows higher variability within and among taxonomic groups (Figure S6). At a global level, we found that 96 species (52%) overlapped with <10% of the coldspots (i.e. OV_coldspot_), whereas only five species (2.7%) overlapped with >50% of the coldspot surface. Species with higher range sizes (e.g. cetaceans) overlapped with a higher proportion of the coldspots (Figure S7a). For example, the proportion of overlap with coldspots ranged from 0% for species with small range sizes, like the royal penguin (*Eudyptes schlegeli*), to values >70% for species with global distribution ranges like the killer whale (*O. orca*) or the humpback whale (*Megaptera novaeangliae*). We found that that 40 species (22%) overlapped >50% of their ranges with coldspots (i.e. OV_range_; Figure S7b). In this case, species with higher range sizes presented lower values of overlap.

### 3.4 Overlap between telemetry data and Argo coldspots

**Figure 5:**
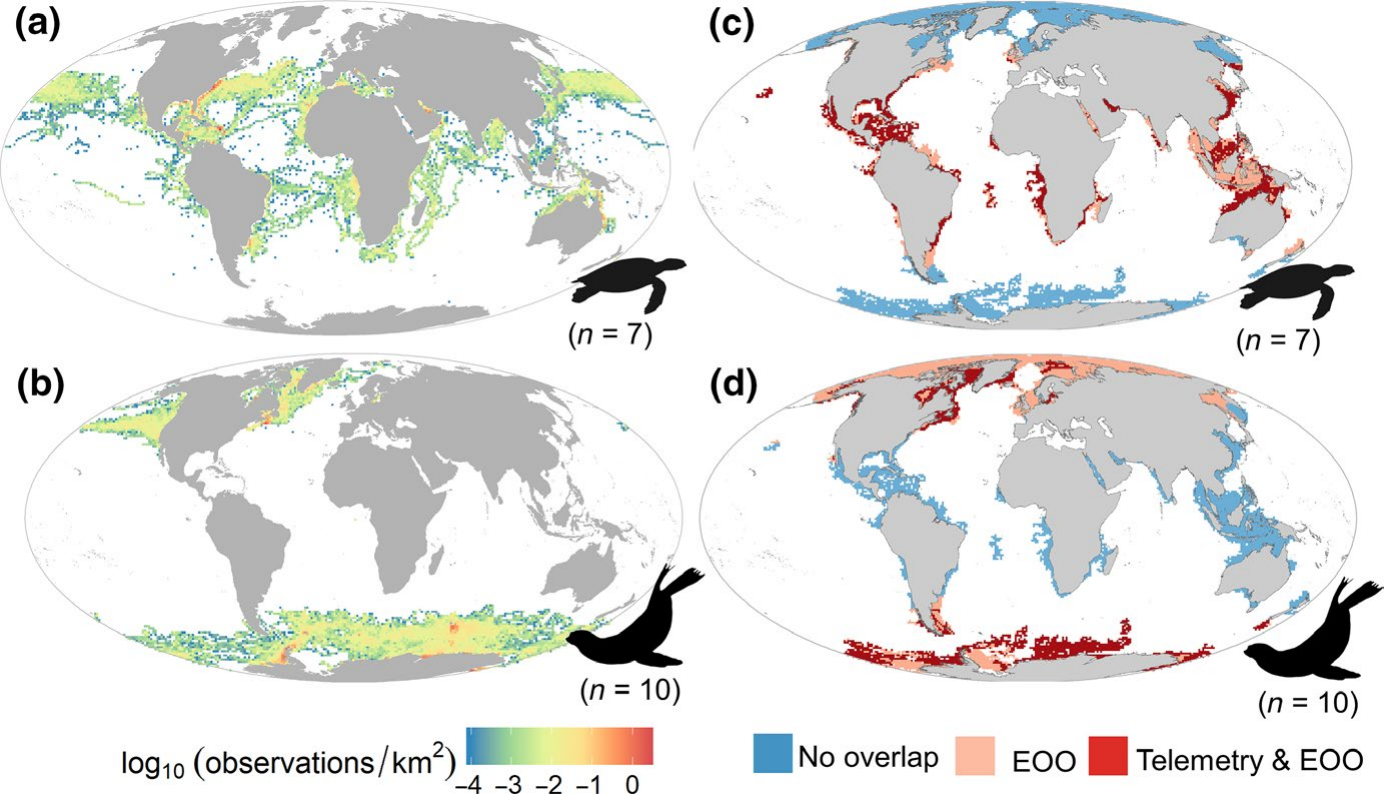
Global density distribution of animal telemetry observations by taxonomic group and their overlap with Argo coldspots. (a) Distribution of sea turtle telemetry records. (b) Distribution of conductivity, temperature, and depth profiles from pinnipeds. Overlap between Argo coldspots, telemetry observations and distribution ranges for sea turtles (c) and pinnipeds (d). Dark red cells represent the overlap of both telemetry observations and distribution ranges with Argo coldspots. Light red cells represent the overlap of only distribution ranges. Numbers between parentheses represent the number of species per taxonomic group

Overlap with satellite tracking data is presented in Figure 5. The restricted patterns of marine turtles and pinnipeds across their latitudinal gradients were consistent between EOO and direct observations from satellite tracking data. Distribution of telemetry observations confirms that pinniped species are currently contributing oceanographic data at higher latitudes, while sea turtle species could complement the Argo network in shallow waters from mid‐ and low latitudes. Telemetry observations of sea turtles overlapped with gaps of the Argo network in key regions of oceanographic interest such as boundary currents (i.e. Gulf stream and Kuroshio current), major upwelling areas (e.g. Canary Current, Benguela Current) and marginal seas (e.g. Caribbean and Indonesian Seas). As expected, telemetry locations were restricted within the ranges defined by the EOO maps and presented a smaller overlap with Argo coldspots (Figure S8a). However, our results show that the restricted areas where the telemetry observations took place present higher proportion of overlap of the species distribution (Figure S8b).

## 4 DISCUSSION

This work synthetizes the distributions of marine species across multiple taxa and assesses overlaps with gaps in oceanographic monitoring at a global scale. By linking Argo coldspots with marine animal distributions, this study provides new insights into using ABI more efficiently to complement OOSs at global and regional scales. While animal‐borne platforms have been used to complement OOSs in polar regions already (Pauthenet et al., 2018; Pellichero et al., 2018; Roquet et al., 2013, 2014), our study particularly highlights the potential contribution in temperate and tropical regions. Such contribution could benefit ocean research in reducing monitoring biases and improving our understanding of key oceanographic processes. Our work has focused on the Argo network due to its global coverage and operational capacity to provide vertical profiles. However, our approach could be extended to other ocean monitoring programmes and could be used to design monitoring networks for other oceanographic platforms.

This work provides a novel framework to assess the gap persistency in ocean monitoring that accounts for long‐term temporal persistence and takes into consideration the spatial coherence of gap surfaces. Unlike previous studies (Cheng et al., 2017; Kuragano, Fujii, & Kamachi, 2015), our approach not only considers under‐sampled regions but also un‐sampled areas, thus allowing the identification of gap areas at a global scale. Here, we provide evidence that significant regions are still under‐sampled and highlight the political challenges in sampling within EEZs. In addition to high latitudes, other larger areas at mid‐latitudes and tropical regions such as the Tropical Asian Archipelago would require additional monitoring efforts. This region represents the largest marginal sea in tropical regions and includes the Indonesian Throughflow, a major area of oceanographic interest (Smith et al., 2019; Von Schuckmann et al., 2014). Overall, coldspot mapping provides an objective way to map gaps in coverage, and produced estimates that are consistent with previous approaches (Kuragano et al., 2015; Roemmich et al., 2019; Von Schuckmann et al., 2014, 2016).

The overlap analysis between marine animals and Argo coldspots reveals areas where ABI could complement global ocean observing strategies. The combination of both EOO maps and tracking observations allowed us to assess the potential and current capabilities of animal‐borne platforms. On the one hand, extant tracking observations provide a good indicator of the current and potential contribution that can be provided by ABI, specifically in pinnipeds and sea turtles. Pinnipeds constitutes the primary group with which oceanographic data have already been collected at higher latitudes and used extensively for oceanographic research (Fedak, 2013; Padman et al., 2010). In temperate and tropical regions, tracking studies of sea turtles confirm their presence in regions of high interest to oceanography (i.e. temperate and tropical coldspots, boundary currents and equatorial upwellings) and environmental data collected by sea turtles have been used for oceanographic purposes in few studies (McMahon et al., 2005; Patel et al., 2018). On the other hand, the overlap with EOO offers new insights into potential contribution from other taxa and species that could provide oceanographic information. Major areas of overlap are concentrated within coastal waters and marginal seas, like the Caribbean Sea or Indonesia Seas. Our results show that some of the largest coldspots areas in temperate and tropical areas align with hotspots of assessed marine species. For example, coastal areas and eastern boundary upwelling systems constitute biological hotspots offering a wide range of foraging habitat to marine megafauna species (Bakun et al., 2015; Rodríguez‐Zárate et al., 2018). Information regarding ocean use by marine fauna in regions such as tropical Asia is still relatively limited (Harcourt et al., 2019). Future studies in these regions offer potential for conservation actions while presenting an important opportunity for cross‐disciplinary collaboration between ecologists and oceanographers.

We have outlined a novel approach for the identification of potential species to fill ocean monitoring gaps of the Argo network at global and regional scales. The high variability in the percentage of cover at the species level reveals the higher heterogeneity of the potential oceanographic collectors within and between taxonomic groups. Species with large ranges (e.g. cetaceans) tend to have a higher percentage of overlap with coldspots. Given the coastal correspondence of coldspots, species with higher coastal affinity (Sequeira et al., 2018) would be more suitable to complement the Argos network. Moreover, other biological traits that may affect data collection and transmission (e.g. body size, depth range or time spent at the surface) would need to be taken into account (Harcourt et al., 2019). For example, air‐breathing animals (e.g. pinnipeds, penguins, sea turtles) present a good balance between vertical profiles and time spent at the surface to relay data via satellite telemetry. Furthermore, differences in life history stage at population or individual levels should need to be taken into account when considering the potential contribution of animal‐borne platforms.

Major gaps in the Argo network with regard to the vertical distribution (i.e. not directly related to the spatial distribution of coldspots) are found in the upper layer (<10 m depth) and great depths (>2,000 m; Von Schuckmann et al., 2016). Air‐breathing taxa can provide profiles of the upper layer on a regular basis. Our results illustrate that cetaceans and pinnipeds constitute the two air‐breathing taxonomic groups that can perform vertical profiles to greater depths (e.g. elephant seals, sperm whales or Cuvier's beaked whales have been observed diving consistently beyond mesopelagic depths of 400–800 m). Some species from other taxonomic groups considered in this study (i.e. sharks and rays, tunas and billfishes) also have the potential to collect environmental information at depths >1,000 m, but with a more limited capacity to provide such information in real‐time as air‐breathing animals. Overall, only eight species (6.6%) presented maximum dive depths greater than 2,000 m (i.e. the profiling target for conventional Argo floats). Animal platforms are, therefore, unlikely to complement the Deep Argo programme, and potential contributions of marine animals to deep sea research would be limited to a few specific cases (Danovaro et al., 2017; Padman et al., 2010).

Problems faced by ABI (e.g. loss of instruments, biofouling and sensor drift) are similar to those faced by other platforms such as gliders or Argo floats. The recovery of oceanographic data by ABI is slightly more challenging in coldspots in the tropics because of the coverage of the ARGOS system (i.e. the main satellite communication system used for wildlife) decreases with decreasing latitude (Jeanniard‐du‐Dot, Holland, Schorr, & Vo, 2017). However, recent advances in the development of new sensors (Nassar et al., 2018), land‐based receiving stations (Jeanniard‐du‐Dot et al., 2017) and communication systems (e.g. 5G networks, or satellite constellations like ARGOS‐4, Icarus, Iridium) offer promising opportunities to expand the range of species and increase the amount and diversity of environmental data collected at reduced costs. In addition, further integration of animal‐borne platforms into open data infrastructures (Block et al., 2016; Treasure et al., 2017), the development of metadata standards (Campbell, Urbano, Davidson, Dettki, & Cagnacci, 2016) and data collection regulations (Kraska, Crespo, & Johnston, 2015; Lennox et al., 2017) will overcome legal and technological barriers and foster the use of ABI for ocean research.

**Figure 6:**
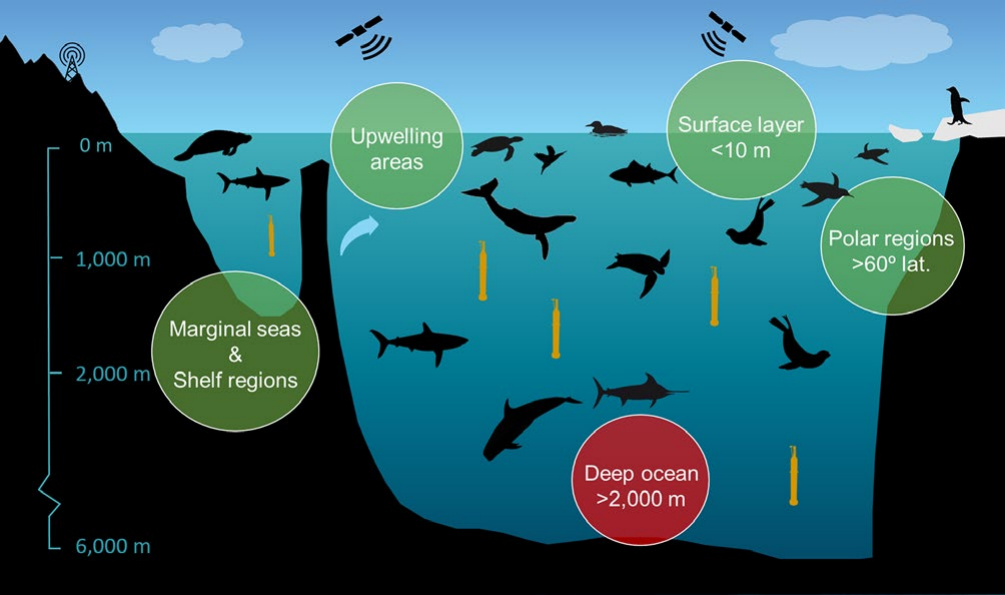
Schematic representation of the gaps of the Argo observing system and potential contribution of animal‐borne instruments. Animal‐borne instruments can contribute in marginal seas, upwelling areas, the upper 10 m of the water column, shelf regions and polewards of 60° latitude. Vertical distribution of taxonomic groups reflects the median and maximum dive depths. Despite some species can dive as much as 2,000 m depth, animal‐borne instruments have limited potential to monitor the deep ocean, which is currently supported by the Deep Argo programme

Our work provides a new foundation to establish, support and improve integrated ocean monitoring programmes, and in particular using ABI, with major implications for global OOSs. Specifically, marine animals could complement observing systems in marginal seas, upwelling areas, the upper 10 m of the water column, shelf regions and polewards of 60° latitude (Figure 6). Therefore, the integration of animal‐borne platforms into the ocean science agenda (Visbeck, 2018) would enhance global progress in combined biological and physical studies to ensure sustainable observations. It is important to note that such programmes should also have clear biological‐oriented objectives and consider welfare and ethical issues. The potential for ABI to help progress our understanding of the global ocean system is, however, very clear.

## CONFLICT OF INTEREST

The authors declare no competing interests. The manuscript was improved as a result of the input of the Editor and two anonymous reviewers.
