# Supplementary material for: Towards the integration of animal‐borne instruments into global ocean observing systems
Source: Glob Chang Biol. 2019 Nov 27;26(2):586–96. doi: 10.1111/gcb.14902 (PMC7027834; doi:10.1111/gcb.14902)
Supplement: Supplementary file 1 [file GCB-26-586-s001.pdf]

Global Change Biology Supporting Information

Towards the integration of animal-borne instruments into global ocean observing systems

David March*, Lars Boehme, Joaquín Tintoré, Pedro Joaquín Vélez-Belchi, Brendan J Godley

***Corresponding author:** David March, email: D.March@exeter.ac.uk. Present address: Centre for Ecology and Conservation, University of Exeter, Cornwall Campus, Penryn TR10 9EZ, United Kingdom.

Citation: March, D. , Boehme, L. , Tintoré, J. , Vélez-Belchi, P. J. and Godley, B. J. (2019), Towards the integration of animal-borne instruments into global ocean observing systems. Glob Change Biol. doi:10.1111/gcb.14902

This PDF includes:

1. Supplementary Material and Methods (Methods S1-S3)
2. Supplementary Tables (S1-S4)
3. Supplementary Figures (S1-S9)

Supplementary Material and Methods

Methods S1. Gap analysis of the Argo network

We used data from the Argo database (>1.5 million profile locations, >13,000 instruments) through the profile directory file of the Argo Global Data Assembly Center (GDAC, accessed on 19th November 2017) (Argo, 2000). We considered the period 2005-2016, given that the Argo network became fully operational with an adequate space-time sampling by 2005 (Von Schuckmann et al., 2016). We counted the number of profiles per year on a regular grid of 1 x 1 degrees (Cheng et al., 2017; Riser et al., 2016). Location data were converted into profile densities (i.e. profiles/km^2). Then, we calculated the coefficient of variation (SD/mean) to assess the inter-annual variation of profile density. Finally, we analysed the geographical distribution of sampling gaps from the Argo network. For each year, we combined un-sampled (i.e. cells without profiles) and under-sampled cells (i.e. cells pertaining to the lowest quintile, <20%, of the profile density distribution) to identify sampling gaps on an annual basis. Then, we calculated the percentage of years a cell was identified as sampling gap and defined persistence gaps those locations with a proportion ≥ 80%. Only spatial coherent gap areas larger than 25 square degrees were retained as coldspot areas (Hobday & Pecl, 2014), eliminating 23% of the total selected grid cells. We used zonal statistics to assess coldspots regions as function of latitude and bathymetry. To assess the political distribution of the Argo coldspots, we summarized them by Exclusive Economic Zones (EEZ) from Marine Regions (http://www.marineregions.org).

Methods S2. Animal-borne platforms

Species list. We combined species lists from previous multi-specific reviews (Hussey et al., 2015; Lascelles et al., 2016; Sequeira et al., 2018) and public marine animal telemetry databases (Halpin et al., 2009; Treasure et al., 2017). Prior to further analysis, the species names were manually inspected for errors. We corrected for misspellings, checked for different common names referring to the same species and aggregated subspecies to the species level. We used online databases (i.e., IUCN, FishBase and SeaLifeBase) to harmonize the taxonomic information and get additional information for each species (e.g., body length).

Distribution ranges. We represented the spatial distribution of the selected animal-borne platform species at a global scale using extent of occurrence maps (EOO) from the International Union for Conservation of Nature (IUCN, 2017). EOO are expert-generated range maps that outline the complete extent of a species' distribution. An alternative to EOO are model-generated species distribution maps that predict the areas occupied by the species (ie. Aquamaps). Despite the trade-offs between approaches, previous studies have found a strong agreement between AquaMaps and IUCN range data for the taxonomic groups analysed in this study (O'Hara, Afflerbach, Scarborough, Kaschner, & Halpern, 2017). EOO maps for seabirds (i.e. flying seabirds and penguins) and turtles were available separately from Bird Life International (BirdLife International, 2017) and the State of the World's Sea Turtles mapping application (Halpin et al., 2009; Kot et al., 2016), respectively. Overall, we identified 183 species of marine vertebrates from the following taxonomic groups: marine turtles (7 species), pinnipeds (28 species), cetaceans (24 species), sirenians (2 species), penguins (10 species), flying seabirds (73 species), tunas and billfishes (11 species), and sharks and rays (29 species). For species with separate subpopulation EOO maps, we combined all subpopulations to create a single global population(O'Hara et al., 2017).

Pinniped satellite tracking data. To being able to compare Argo float coverage with the potential of environmental data from animal-borne instruments, we excluded the full resolution location data from the behavioural studies and used data from the MEOP-CTD database (Treasure et al., 2017). This database contained a collection of hydrographic profiles (>800,000) from CTDs mounted on marine mammals (>1,000 individuals). We selected all profiles recorded during the period 2004-2017. We counted the number of profiles on a regular grid of 1 x 1 degrees per species and all species pooled. Count data were converted into profile densities (e.g. profiles per km^2). After correcting for misspellings and checking for different common names referring to the same species, we conducted our analysis for the 10 species of pinnipeds available in the database (Table S4).

Turtle satellite tracking data. We used a product from OBIS-SEAMAP (Halpin et al., 2009) (accessed on 23rd March 2017) that summarized the number of telemetry records on a regular grid of 0.1 x 0.1 degrees for each of the seven species. The dataset included 893,810 records from 2,101 sea turtles tagged between 1993-2017 (Table S4). We filtered

out grid cells located on land polygons using the 1:50 vector map from Natural Earth (www.naturalearthdata.com), resampled at 1 x 1 degrees and calculated the total number of records per species for each cell. Count data was converted into records densities (e.g. records per km^2). In order to assess potential biases of telemetry data, we compared the OBIS-SEAMAP database with a previous study that analysed the distribution of sea turtle telemetry studies by species and ocean regions(Jeffers & Godley, 2016). In order to summarize the distribution of species per ocean regions, we overlapped the bounding area of each tagged animal with the ocean regions from the "The SeaVoX Salt and Fresh Water Body Gazetteer" (v16 2015). Comparison in the proportion of animals tracked and studies per ocean regions are presented in Figure S9.

Methods S3. Data projection and representation.

The area of analysis was limited to ocean cells based on land polygons from Natural Earth and the gridded bathymetry from the General Bathymetric Chart of the Oceans (GEBCO, 2014). Grid cells from the Caspian Sea and with <10% ocean area were removed from the analysis (Tittensor et al., 2010). Because of our resampling to 1° resolution, absolute surface values should be taken with care. However, compared with previous works, our method overestimates <0.04% previous sea surface estimates (Costello, 2010).

References

Argo. (2000). Argo float data and metadata from Global Data Assembly Centre (Argo GDAC). https://doi.org/http://doi.org/10.17882/42182

BirdLife International. (2017). BirdLife International and Handbook of the Birds of the World (2017) Bird species distribution maps of the world. Version 2017.2. Available at http://datazone.birdlife.org/species/requestdis.

Cheng, L., Trenberth, K. E., Fasullo, J., Boyer, T., Abraham, J., & Zhu, J. (2017). Improved estimates of ocean heat content from 1960 to 2015. *Science Advances*, *3*(3), 1–11. https://doi.org/10.1126/sciadv.1601545

Costello, M. J. (2010). Surface Area and the Seabed Area, Volume, Depth, Slope, and Topographic Variation for the World's Seas, Oceans, and Countries. *Environmental Science & Technology*, *44*(23), 8821–8828. https://doi.org/10.1021/es1012752

GEBCO. (2014). The GEBCO 2014 Grid, version 20150318, www.gebco.net.

Halpin, P., Read, A., Fujioka, E., Best, B., Donnelly, B., Hazen, L., … Hyrenbach, K. D.

(2009). OBIS-SEAMAP: The World Data Center for Marine Mammal, Sea Bird, and Sea Turtle Distributions. *Oceanography, 22*(2), 104–115. https://doi.org/10.5670/oceanog.2009.42

Hobday, A. J., & Pecl, G. T. (2014). Identification of global marine hotspots: Sentinels for change and vanguards for adaptation action. *Reviews in Fish Biology and Fisheries, 24*(2), 415–425. https://doi.org/10.1007/s11160-013-9326-6

Hussey, N. E., Kessel, S. T., Aarestrup, K., Cooke, S. J., Cowley, P. D., Fisk, A. T., … Whoriskey, F. G. (2015). Aquatic animal telemetry: A panoramic window into the underwater world. *Science*, *348*(6240), 1255642. https://doi.org/10.1126/science.1255642

IUCN. (2017). The IUCN Red List of Threatened Species. Version 2017-3. http://www.iucnredlist.org.

Jeffers, V. F., & Godley, B. J. (2016). Satellite tracking in sea turtles: How do we find our way to the conservation dividends? *Biological Conservation, 199,* 172–184. https://doi.org/10.1016/j.biocon.2016.04.032

Kot, C. Y., E. Fujioka, A.D. DiMatteo, B.P. Wallace, Hutchinson, B. J., Cleary, J., … R.B. Mast. (2016). The State of the World’s Sea Turtles Online Database: Data provided by the SWOT Team and hosted on OBIS-SEAMAP. Oceanic Society, Conservation International, IUCN Marine Turtle Specialist Group (MTSG), and Marine Geospatial Ecology Lab, Duke University. Retrieved from http://seamap.env.duke.edu/swot

Lascelles, B. G., Taylor, P. R., Miller, M. G. R., Dias, M. P., Oppel, S., Torres, L., … Small, C. (2016). Applying global criteria to tracking data to define important areas for marine conservation. *Diversity and Distributions, 22*(4), 422–431. https://doi.org/10.1111/ddi.12411

O’Hara, C. C., Afflerbach, J. C., Scarborough, C., Kaschner, K., & Halpern, B. S. (2017). Aligning marine species range data to better serve science and conservation. *PLoS ONE*, *12*(5), 1–15. https://doi.org/10.1371/journal.pone.0175739

Riser, S. C., Freeland, H. J., Roemmich, D., Wijffels, S., Troisi, A., Belbeoch, M., … Jayne, S. R. (2016). Fifteen years of ocean observations with the global Argo array. *Nature Clim. Change*, *6*(2), 145–153. https://doi.org/10.1038/nclimate2872

Sequeira, A. M. M., Rodríguez, J. P., Eguíluz, V. M., Harcourt, R., Hindell, M., Sims, D. W., … Thums, M. (2018). Convergence of marine megafauna movement patterns in coastal and open oceans. *Proceedings of the National Academy of Sciences, 115*(12),

3072–3077. https://doi.org/10.1073/pnas.1716137115

Tittensor, D. P., Mora, C., Jetz, W., Lotze, H. K., Ricard, D., Berghe, E. Vanden, & Worm, B. (2010). Global patterns and predictors of marine biodiversity across taxa. *Nature*, *466*(7310), 1098–1101. https://doi.org/10.1038/nature09329

Treasure, A., Roquet, F., Ansorge, I., Bester, M., Boehme, L., Bornemann, H., … de Bruyn, P. J. N. (2017). Marine Mammals Exploring the Oceans Pole to Pole: A Review of the MEOP Consortium. *Oceanography*, *30*(2), 132–138. https://doi.org/10.5670/oceanog.2017.234

Von Schuckmann, K., Palmer, M. D., Trenberth, K. E., Cazenave, A., Chambers, D., Champollion, N., … Wild, M. (2016). An imperative to monitor Earth's energy imbalance. *Nature Climate Change*, *6*(2), 138–144. https://doi.org/10.1038/nclimate2876

Supplementary Tables

Table S1. Surface estimates of Argos coldspots at a global and regional scales.

Region	Ocean area (x10^4 km^2)	Coldspot area (x10^4 km^2)	Coldspot inside EEZ (%)	No. of species	$OV_{coldspot}$ (mean ± SD)	OV_{range} (mean ± SD)
Northern Ocean (30ºN-60ºN]	5,070	924	89.8	120	0.20 ± 0.20	0.25 ± 0.24
Tropical Ocean [30ºS-30ºN]	19,559	2,533	93.5	150	0.27 ± 0.34	0.21 ± 0.23
Southern Ocean (30ºS-60ºS]	8,822	652	66.3	135	0.28 ± 0.30	0.18 ± 0.23
Artic Ocean (60ºN-90ºN]	1,889	1,600	71.6	58	0.14 ± 0.18	0.62 ±0.26
Antarctic Ocean [90ºS-60ºS)	2,103	1,266	43.3	48	0.42 ± 0.38	0.45 ± 0.17
Global Ocean [90ºS-90ºN]	37,443	6,975	76.3	183	0.15 ± 0.15	0.26 ± 0.24

Table S2. Surface estimates of Argos coldspots of the top 15 EEZ.

EEZ	Coldspot area ($x10^4$ km^2)	EEZ area ($x10^4$ km^2)
**Russia**	652.1 (12.2%)	777.2
**Antarctica**	539.4 (10.1%)	962.5
**Canada**	525.9 (9.9%)	578.3
**Indonesia**	511.2 (9.6%)	602.8
**United States**	310.2 (5.8%)	1,216.5
**Australia**	277.3 (5.2%)	903.0
**Mexico**	180.2 (3.4%)	319.0
**United Kingdom**	147.2 (2.8%)	662.2
**Denmark**	133.3 (2.5%)	263.5
**Norway**	128.9 (2.4%)	246.0
**Brazil**	119.4 (2.2%)	368.1
**Chile**	104.5 (2.0%)	366.3
**China**	93.6 (1.8%)	96.5
**Argentina**	85.7 (1.6%)	107.3
**New Zealand**	85.0 (1.6%)	671.5
**All EEZ**	5324.8 (100%)	1,5104.6

Table S3. Summary of biological traits and overlap indices per taxonomic group. Values correspond to mean ± SD, except for the maximum dive depths that correspond to median (range). Summaries for body length and maximum dive depths were computed with a different number of species for several groups due to data availability. In those cases, the number of individuals used is annotated between parentheses. Overlap indices correspond to averaged values at global level.

**Group**	**Num. species**	**Range size ($x10^4$ km^2)**	**Body length (m)**	**Maximum dive depth (m)**	**$OV_{coldspot}$**	**OV_{range}**
Cetacean	24	16,180 ± 13,708	9.9 ± 8.9 (23)	647 (58.5 – 3,510) (21)	0.33 ± 0.25	0.29 ± 0.26
Flying bird	73	6,214 ± 4,762	0.7 ± 0.4 (51)	12.4 (0.6 – 140) (17)	0.09 ± 0.08	0.14 ± 0.14
Penguin	10	1,256 ± 1,052	0.7 ± 0.2	171.5 (69 – 564)	0.06 ± 0.06	0.36 ± 0.23
Pinniped	27	1,293 ± 1,454	2.7 ± 1.1 (25)	388 (105 – 2,149)	0.08 ± 0.08	0.46 ± 0.27
Shark and ray	29	9,113 ± 10,024	5.2 ± 3.5	440 (22 – 2,200) (28)	0.21 ± 0.14	0.36 ± 0.23
Sirenian	2	626 ± 559	4.0 ± 0.1	20.25 (20 – 20.5)	0.06 ± 0.05	0.68 ± 0.09
Tuna and Billfish	11	16,359 ± 7,993	3.6 ± 1.0	464 (15 – 2,878)	0.20 ± 0.10	0.09 ± 0.03
Turtle	7	11,139 ± 8,008	1.2 ± 0.7 (6)	185.5 (11 – 1,250)(6)	0.23 ± 0.14	0.30 ± 0.28

Table S4. Summary of tracking data for pinniped and turtle species. Overlap indices correspond to global level.

**Species**	**Animals tagged**	**Number of observations**[1]	**Range ($x10^4$ km^2)**	OV_{range}	$OV_{coldspot}$
**Pinniped**					
Cystophora cristata	33	21,895	522	0.456	0.034
Erignathus barbatus	5	202	24	1	0.003
Halichoerus grypus	55	37,479	115	0.852	0.014
Hydrurga leptonyx	7	2,940	4	0.5	0
Leptonychotes weddellii	156	55,768	100	0.86	0.012
Lobodon carcinophaga	9	4182	40	0.925	0.005
Mirounga angustirostris	88	22,877	719	0.047	0.005
Mirounga leonina	581	262,949	4779	0.249	0.171
Pagophilus groenlandicus	19	4,607	258	0.698	0.026
Pusa hispida	53	11,642	152	0.987	0.022
**Turtle**					
Caretta caretta	951	461,233	4467	0.159	0.102
Chelonia mydas	385	152,296	1331	0.48	0.092
Dermochelys coriacea	62	36,256	1431	0.154	0.032
Eretmochelys imbricata	345	209,433	604	0.611	0.053
Lepidochelys kempii	133	43,344	66	0.909	0.009
Lepidochelys olivacea	103	39,995	981	0.32	0.045
Natator depressus	122	54,818	148	0.865	0.018

[1] Number of observations refers to “profiles” for pinnipeds, and “records” for sea turtles.

Supplementary Figures

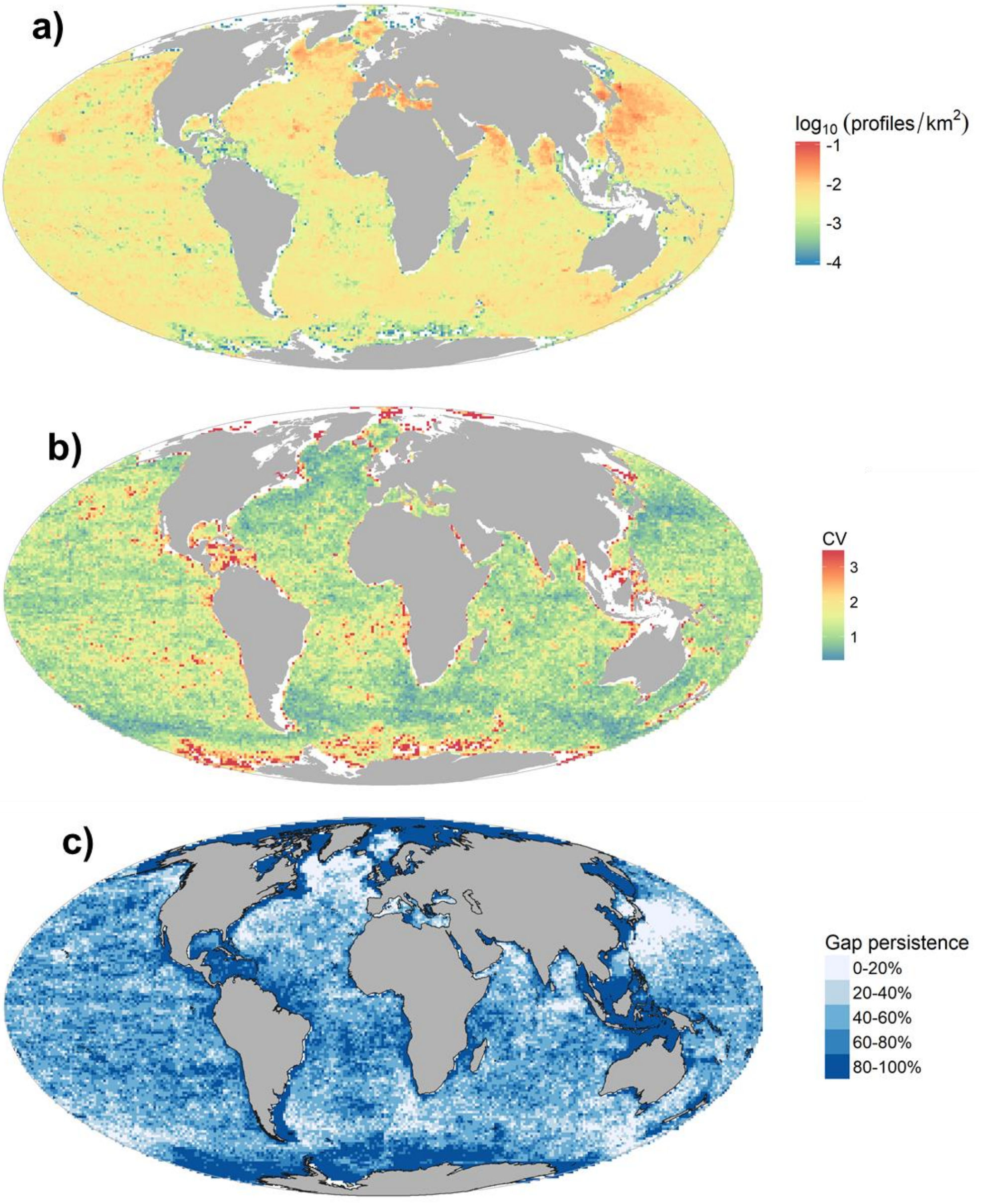

Figure S1. Global patterns of the spatial distribution of the Argo network for the period 2005-2016. **(a)** Density distribution of Argo profiles. **(b)** Coefficient of variation (CV) of the annual distribution of vertical profiles. Higher values indicate areas of greatest variability in monitoring efforts. **(c)** Persistence of gap areas. In plots a-b, white cells correspond to cells where no profiles were performed.

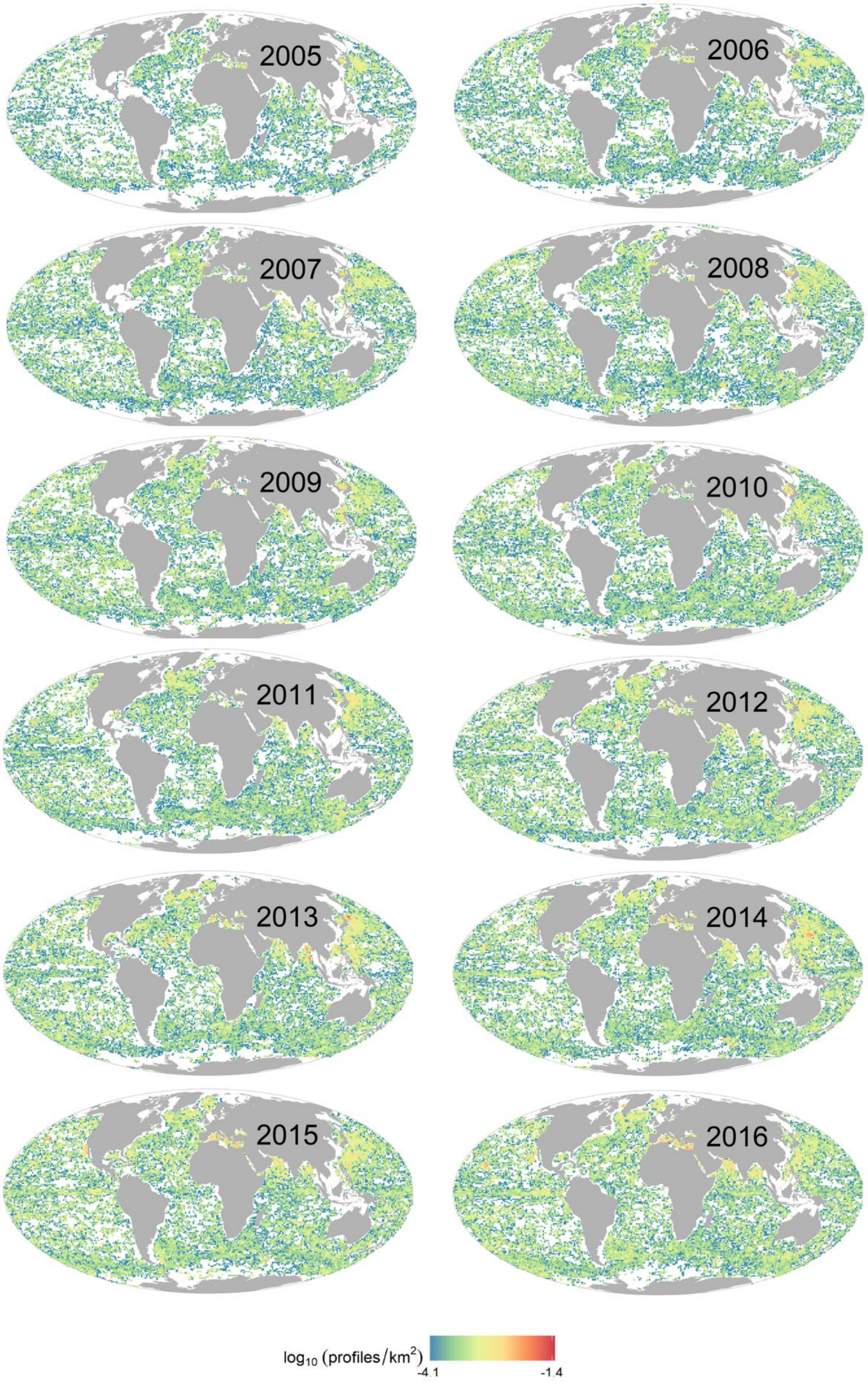

Figure S2. Annual density distribution of CTD profiles from the Argo network for the period 2005-2016. White cells correspond to cells where no profiles were performed.

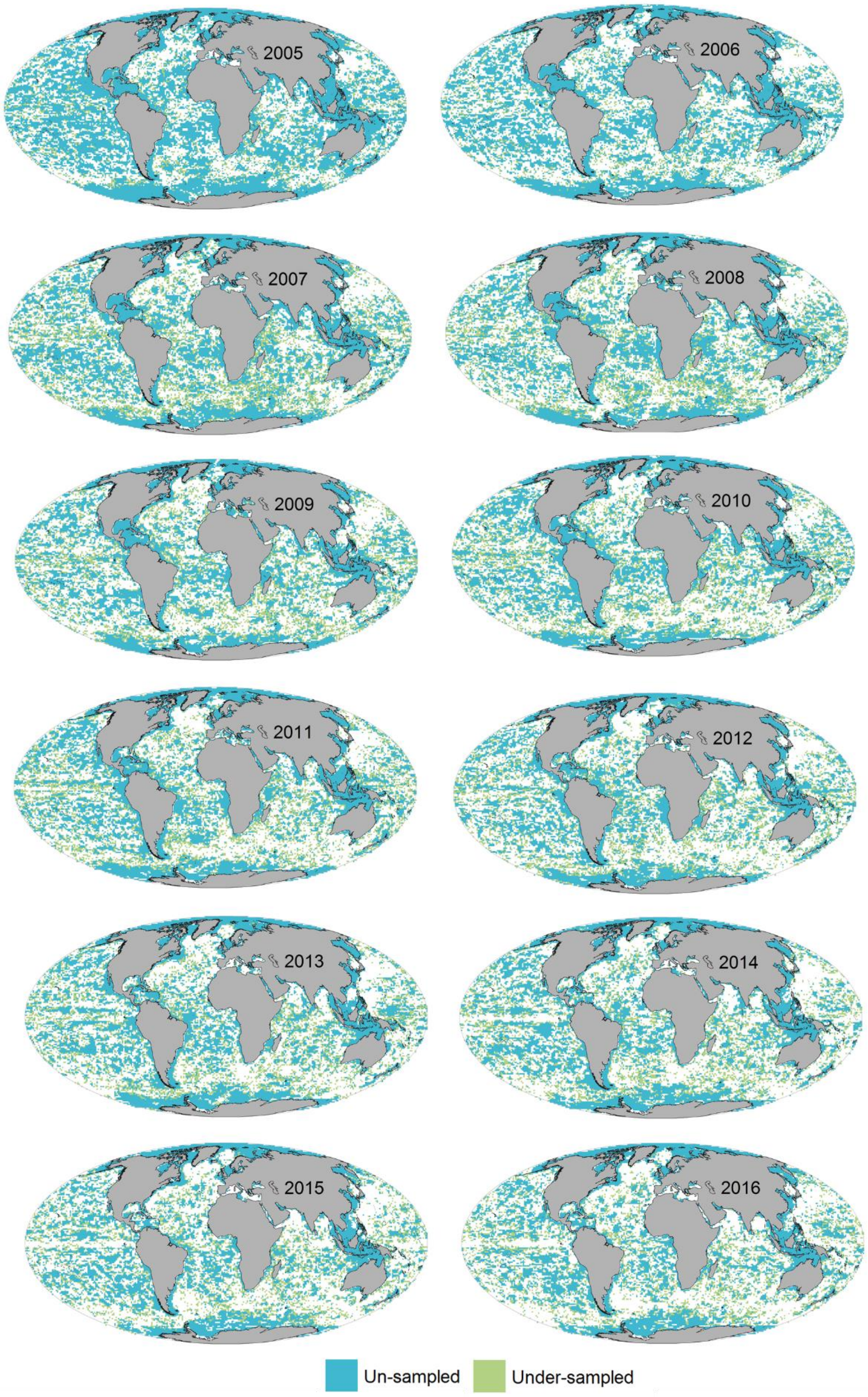

Figure S3. Spatial distribution of un-sampled (blue) and under-sampled areas (green) from the Argo network for the period 2005-2016 on an annual basis. White cells correspond to areas that were monitored by Argos profiles.

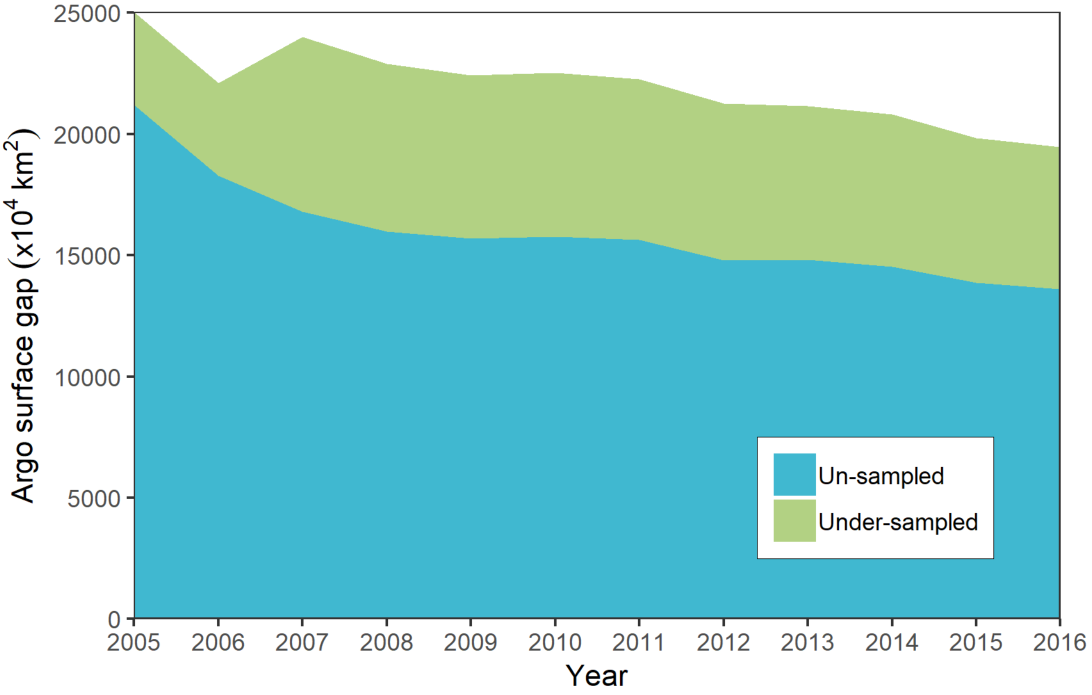

Figure S4. Temporal distribution of sampling gap surface of the Argo network for the period 2005-2016 on an annual basis. Sampling gaps considered both un-sampled regions (i.e. areas without profiles) and under-sampled regions (i.e. areas pertaining to the lowest quintile, <20%, of the Argo profile density distribution).

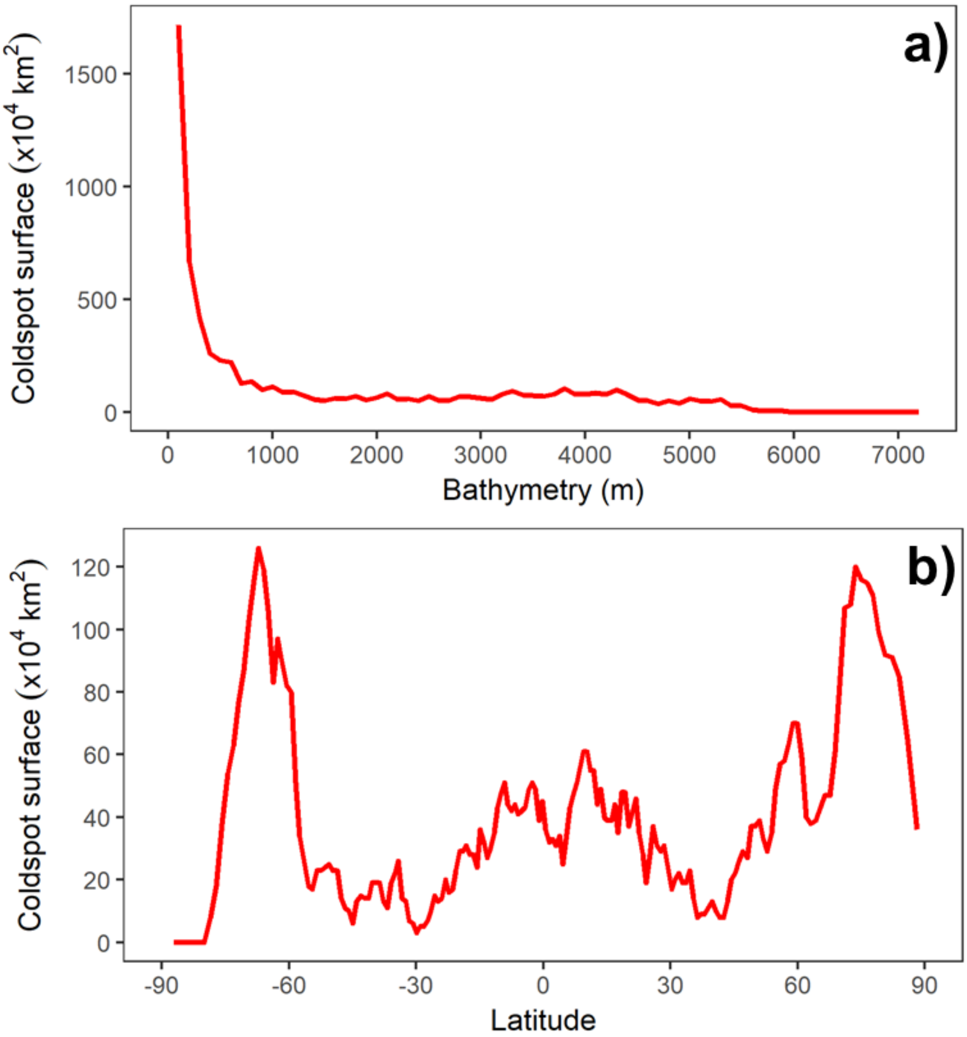

Figure S5. Distribution of the Argo coldspot areas across bathymetric (**a**) and latitudinal (**b**) gradients.

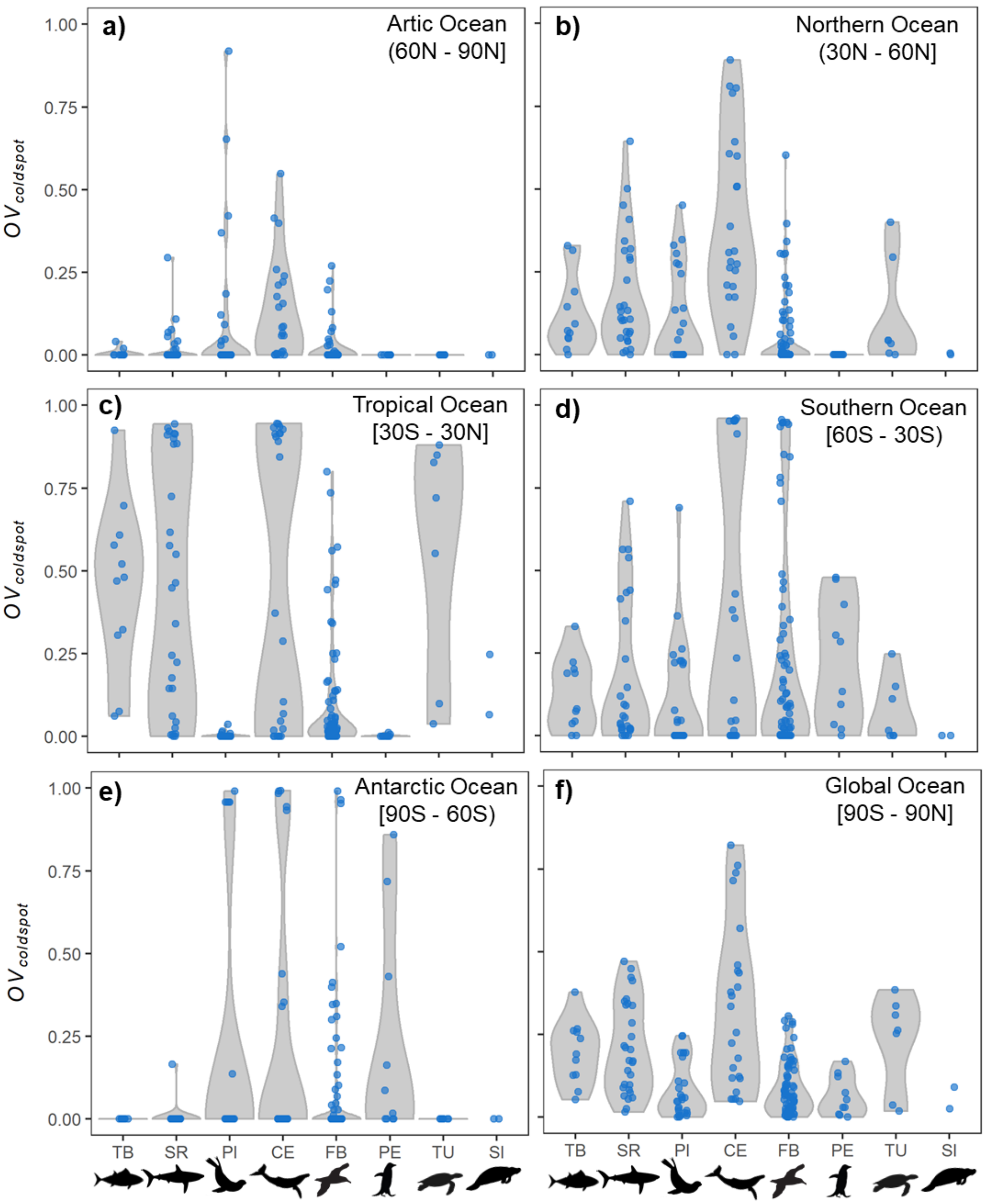

Figure S6. Violin plots detailing the spatial overlap between the gap of the Argo network and animal-borne platforms species by taxonomic group and region. Points in blue represent selected species.

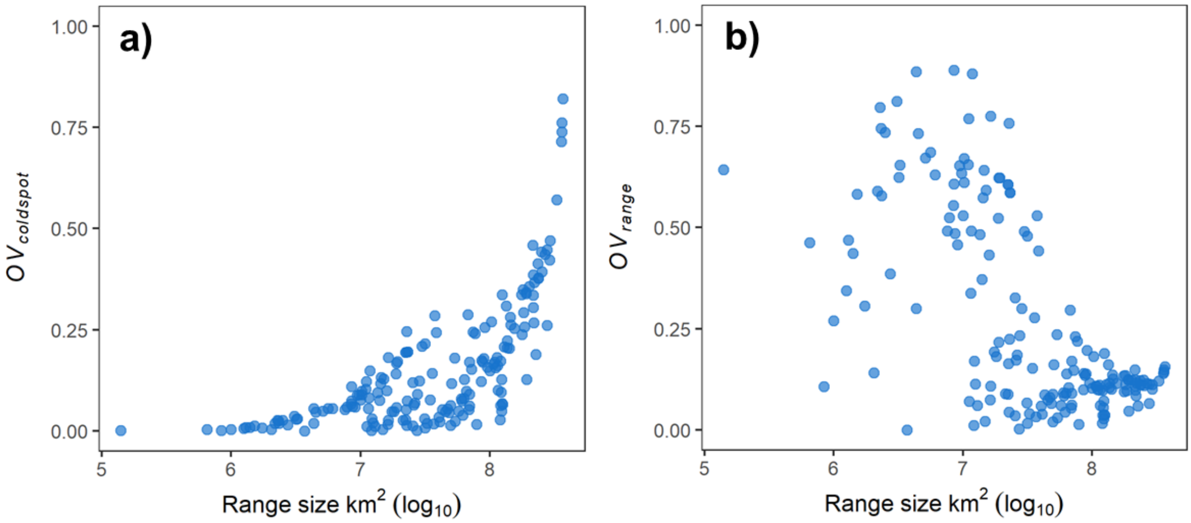

Figure S7. Relationship between species range size and overlap indices**: (a)** percentage of coldspots overlapped by species ranges, **(b)** percentage of species ranges overlapped by coldspots. Overlap indices correspond to global level.

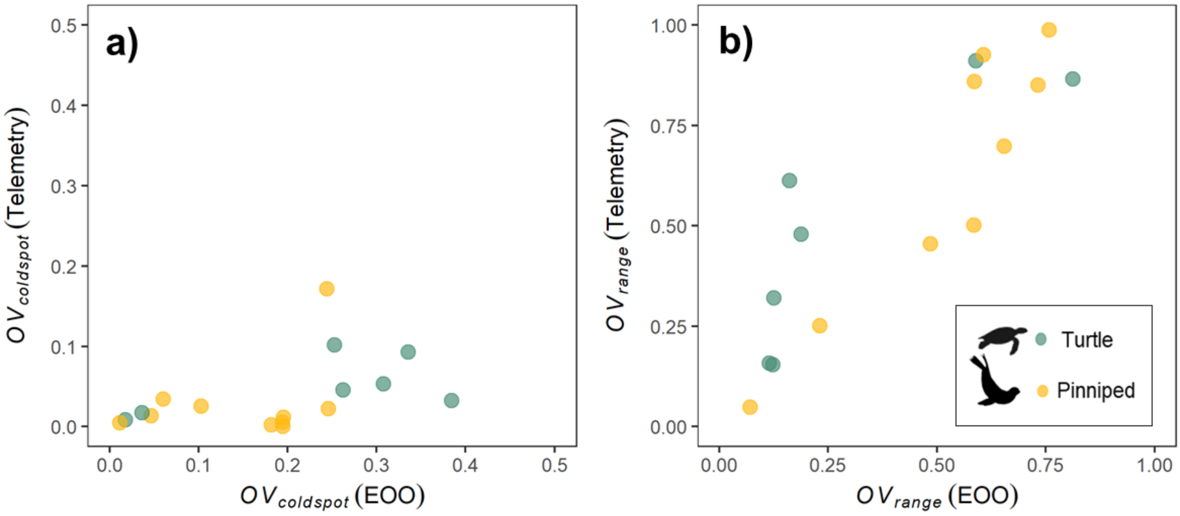

Figure S8. Comparison between overlap indices and data sources (telemetry and EOO ranges) for turtles and pinnipeds. Overlap indices correspond to global level.

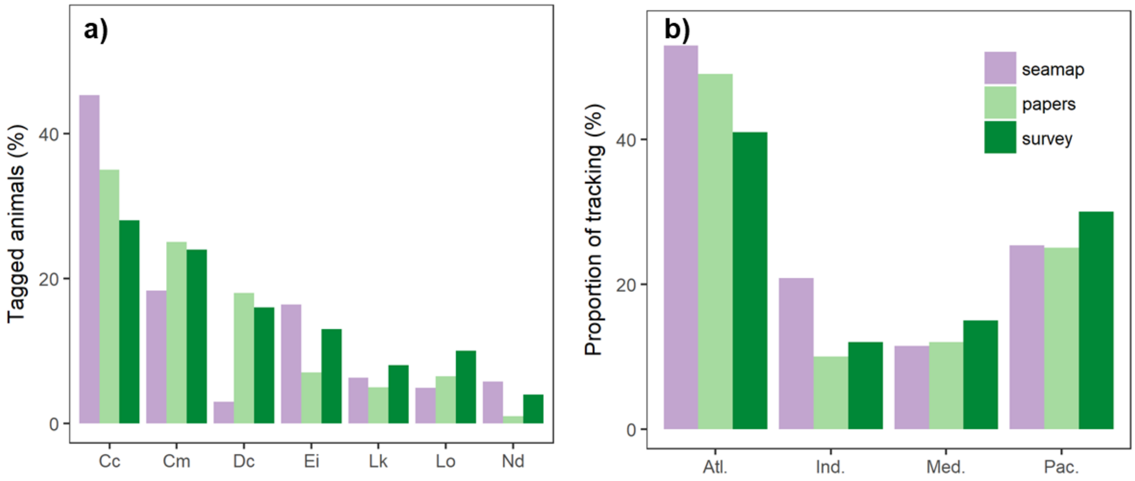

Figure S9. Assessment of the bias in sea turtle tracking data. Comparison between OBIS-SEAMAP dataset with Jeffers and Godley (2015). (**a**) Proportion of tagged species. Cc, *Caretta caretta*; Cm, *Chelonia mydas*; Dc, *Dermochelys coriacea*; Ei, *Eretmochelys imbricate*; Lk, *Lepidochelys kempii*; Lo, *Lepidochelys olivacea*; Nd, *Natator depressus*. (**b**) Proportion of tagged animals and telemetry studies per ocean regions. Atl, Atlantic Ocean; Ind, Indian Oceans; Med, Mediterranean Sea; Pac, Pacific Ocean.
